# Open LLM-based actionable incidental finding extraction from [^18^F]fluorodeoxyglucose PET-CT radiology reports

**DOI:** 10.3389/fdgth.2025.1702082

**Published:** 2025-11-14

**Authors:** Stephen H. Barlow, Sugama Chicklore, Yulan He, Sebastien Ourselin, Thomas Wagner, Anna Barnes, Gary J. R. Cook

**Affiliations:** 1 School of Biomedical Engineering and Imaging Sciences, King’s College London, London, United Kingdom; 2 King’s College London and Guy’s and St. Thomas’ PET Centre, St. Thomas’ Hospital, London, United Kingdom; 3 Department of Informatics, King’s College London, London, United Kingdom; 4 Department of Computer Science, University of Warwick, Coventry, United Kingdom; 5 Alan Turing Institute, London, United Kingdom; 6 Department of Nuclear Medicine, Royal Free Hospital, London, United Kingdom; 7 Department of Imaging, Centre for Medical Imaging, University College London, London, United Kingdom; 8 King’s Technology Evaluation Centre (KiTEC), School of Biomedical Engineering & Imaging Science, King’s College London, London, United Kingdom

**Keywords:** incidental findings, natural language processing, diagnostic imaging, artificial intelligence, positron emission tomography-computed tomography

## Abstract

**Introduction:**

We developed an open, large language model (LLM)-based pipeline to extract actionable incidental findings (AIFs) from [^18^F]fluorodeoxyglucose positron emission tomography-computed tomography ([^18^F]FDG PET-CT) reports. This imaging modality often uncovers AIFs, which can affect a patient's treatment. The pipeline classifies reports for the presence of AIFs, extracts the relevant sentences, and stores the results in structured JavaScript Object Notation format, enabling use in both short- and long-term applications.

**Methods:**

Training, validation, and test datasets of 1,999, 248, and 250 lung cancer [^18^F]FDG PET-CT reports, respectively, were annotated by a nuclear medicine physician. An external test dataset of 460 reports was annotated by two nuclear medicine physicians. The training dataset was used to fine-tune an LLM using QLoRA and chain-of-thought (CoT) prompting. This was evaluated quantitatively and qualitatively on both test datasets.

**Results:**

The pipeline achieved document-level F1 scores of 0.917 ± 0.016 and 0.79 ± 0.025 on the internal and external test datasets. At the sentence-level, F1 scores of 0.754 ± 0.011 and 0.522 ± 0.012 were recorded, and qualitative analysis demonstrated even higher practical utility. This qualitative analysis revealed how sentence-level performance is better in practice.

**Discussion:**

Llama-3.1-8B Instruct was the base LLM that provided the best combination of performance and computational efficiency. The utilisation of CoT prompting improved performance further. Radiology reporting characteristics such as length and style affect model generalisation.

**Conclusion:**

We find that a QLoRA-adapted LLM utilising CoT prompting successfully extracts AIF information at both document- and sentence-level from both internal and external PET-CT reports. We believe this model can assist with short-term clinical challenges like clinical alerts and reminders, and long-term tasks like investigating comorbidities.

## 1 Introduction

[^18^F]Fluorodeoxyglucose positron emission tomography-computed tomography (FDG PET-CT) is a medical imaging modality used extensively in cancer treatment (1). It frequently reveals actionable incidental findings (AIFs) (2), medical phenomena, separate from the reason for the scan, requiring clinical intervention or observation (3). Strategies for AIF management are a focus of wider study (4), and decision support systems utilising LLMs could benefit these efforts. Distinguishing between AIFs and other incidental findings is important for prioritising resources, developing a richer patient assessment, and improving patient wellbeing (3). Real-time AIF extraction could also ensure that appropriate action is taken promptly and preserve AIF details for comorbidity investigations later in a patient's health journey.

Large language models (LLMs) have been shown to successfully extract clinical information from free text (5). Furthermore, parameter-efficient fine-tuning techniques, such as low-rank adaptation (LoRA) (6), prompt engineering techniques such as chain-of-thought (CoT) prompting (7), and quantisation techniques allow publicly available LLMs to be adapted to domain-specific tasks (8), even in resource-constrained environments.

Work has been done to extract incidental findings from radiology reports (9–12), but less for PET-CT specifically, where only one study attempting to extract ‘secondary findings’ alongside primary cancers was found (13). The methodologies in these studies differ. The earliest study found by Dutta et al. (9) utilised a rule-based approach to determine whether further imaging was required for incidental findings from a range of imaging modalities. Evans et al. (10) classified reports at the document level for the presence or absence of incidental findings using a random forest model. Trivedi et al. (11) used word and concept embeddings alongside various classification approaches to identify incidental findings at both the section and sentence levels. Woo et al. (12) utilised GPT-4 (14) to locate ‘definitely actionable’ and ‘possibly actionable’ incidental findings from x-ray, CT, and ultrasound scans. GPT-4 and similar proprietary LLMs can pose a risk both to patient privacy and methodological rigour. This is because patient data leaves hospital servers to be processed by OpenAI, whose lack of public version control jeopardises reproducibility (15). Developing alternatives with open LLMs is important for broader implementation in clinical practice. Accordingly, we developed an open large language model (LLM)-based pipeline to extract AIFs from PET-CT reports. It automatically classifies reports for the presence of AIFs, extracts the relevant sentences, and outputs the results in structured JavaScript Object Notation (JSON) format, enabling use in both short- and long-term applications. This provides an open LLM-based alternative to Woo et al.'s (12) closed-source approach and represents the first work found to extract AIFs [as opposed to ‘secondary findings’ (13)] from PET-CT reports.

## 2 Materials and methods

### 2.1 Clinical data

The PET-CT report dataset used in this study was created in an earlier study (16) and consisted of an *internal* dataset from King's College London and Guy's and St Thomas' PET Centre and an *external* test set from the Royal Free Hospital. The internal reports were from 2012 to 2021, and the external reports are from 2020. The training, validation, and test splits were kept from the earlier study except for removing one validation set report (where lung cancer was found to be an AIF and not the reason for the scan) (16). This research was developed with Guy's Cancer Cohort (ref: 18/NW/0297), and accordingly, the data use was approved by a UK Research Ethics Committee (UK IRAS 228790) (17).

Guidelines to define AIFs were developed using resources from both the American College of Radiology and the Royal College of Radiologists (18–20). As these are general guidelines, developed with multiple imaging modalities in mind, some inclusion/exclusion criteria were adapted to be more suitable for PET-CT scans for lung cancer, as the actionability of an incidental finding is not an absolute characteristic but determined by the wider status of a patient's health (21). An example is emphysema, which is common in lung cancer patients (22). Given the clinical context of a lung cancer patient, it would be included when an intensifying qualifier was used in conjunction with it (such as ‘severe’) but excluded when diminishing or neutral qualifications were used (‘mild’, ‘moderate’). Other examples of AIFs include abdominal aortic aneurysms and other incidental non-pulmonary malignancies.

We used a two-stage annotation approach, where the reports were initially annotated by either one (GC—internal data) or two (GC and SC—external data) expert annotators with 30 and 14 years of PET experience, respectively. Two expert annotators were used on the external data to test inter-annotator agreement. Any disagreements between the two annotators on the external data were resolved before the second annotation stage. In the second stage, SB verified the annotations by error-catching missed findings. For example, both annotators may have agreed on a finding in the ‘Interpretation’ section of the report but missed another reference to the same finding in the ‘Findings’ section. Whenever a missed finding was found, it was checked with the clinical annotators. This process maximised annotation accuracy while using expert time efficiently.

**Figure 1:**
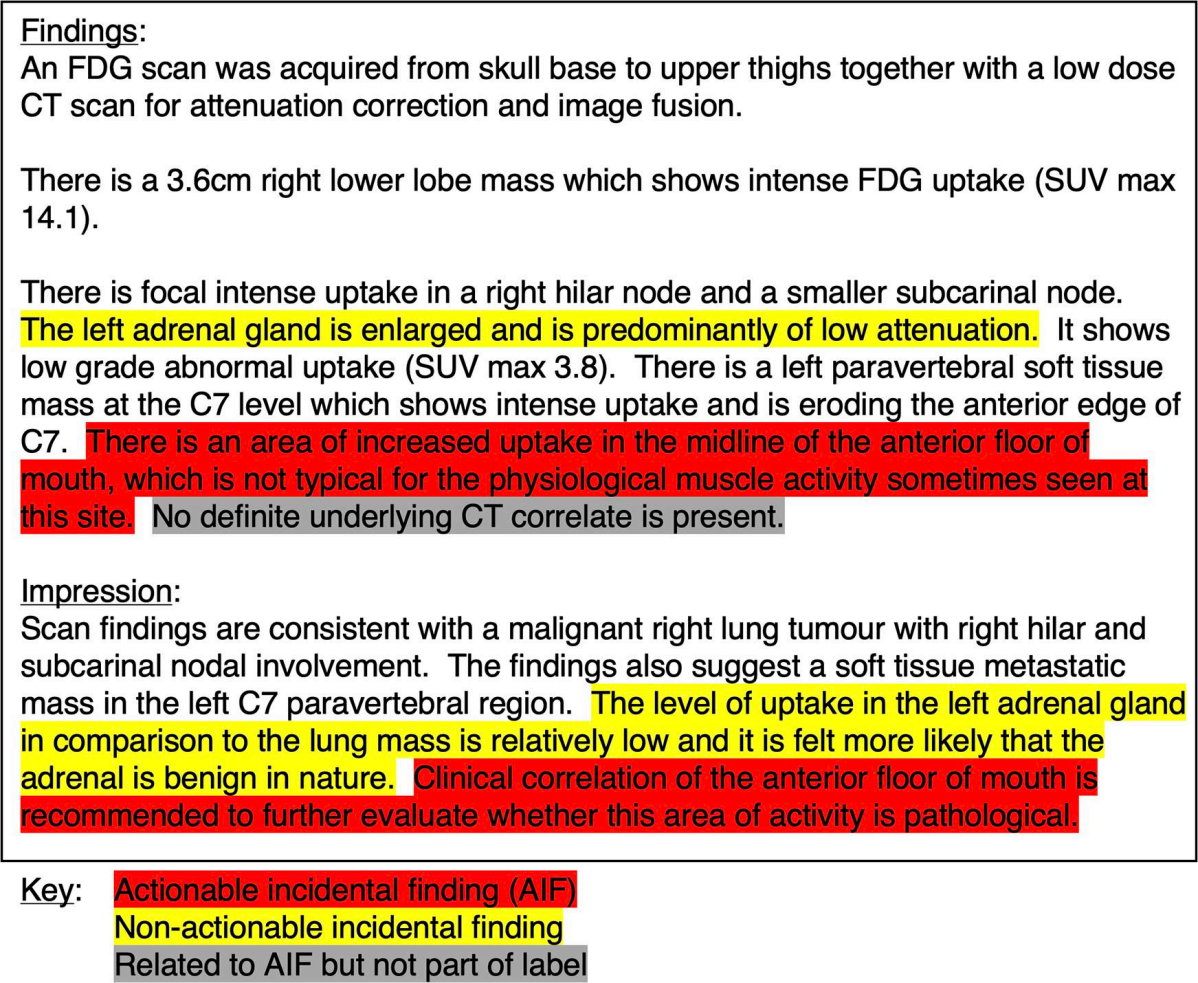
Example PET-CT report with highlighted text distinguishing between different types of incidental finding sentences.

Following annotation, the internal and external data were analysed to observe if differences in reporting style could be quantified (16). We investigated the document-level class distributions, the number of tokens per report (using Llama 3.1's tokenizer), and the number of AIF sentences per document. Figure 1 shows an example report with annotations.

### 2.2 LLMs

LLMs are computationally expensive and beyond the resources of most hospitals. This creates issues as patient data is confidential, often requiring model development to be performed on-site. Using open LLMs mitigates this concern while offering greater replicability, both for research and clinical validation. Accordingly, no proprietary LLMs would be used, and the LLMs used must be trainable on consumer-level equipment. The graphics processing unit (GPU) used in this project was an NVIDIA GeForce RTX 3090. This is still unlikely to be available to most UK hospitals, but it has the potential to be achieved locally. Models from the Llama, Phi, Gemma, and Mistral families of LLMs were tested with parameter counts ranging from 1 to 14 billion (23–28). Due to our small fine-tuning dataset, we used the instruction-tuned variants of each LLM to benefit from the additional training these have undergone. Additionally, we trialled Saama's OpenBioLLM-Llama3-8B to test if using an LLM that has undergone further medical domain adaptation improves performance (29). The fine-tuning objective was next-token prediction on the prompt, report, and desired output for each training example. Finally, we trained a binary sentence classification model using GatorTron [a 355 million-parameter Bidirectional encoder representations from transformers (BERT)-style model shown to perform well on PET-CT reports] to serve as a non-generative baseline (16, 30).

### 2.3 Prompting

**Figure 2:**
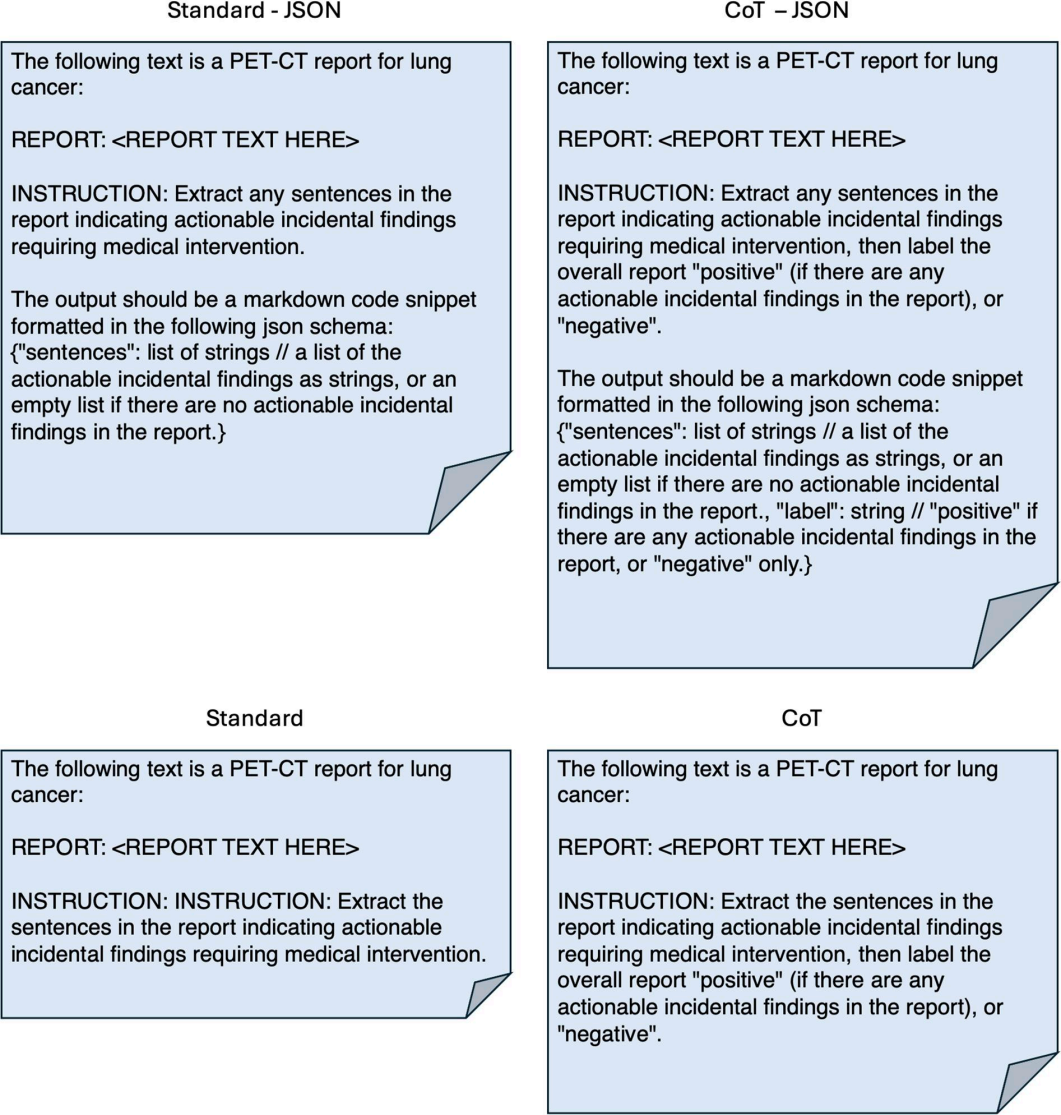
The four prompt templates used for training the model. <REPORT TEXT HERE> represents where the text of each PET-CT report would be inserted into the prompt before tokenization and being inputted to the model.

The format of the instruction given to an LLM has been shown to impact the quality and reliability of responses (7, 31). Accordingly, we experimented with four prompt templates: ‘Standard—JSON’, ‘CoT—JSON’, ‘Standard', and ‘CoT’. Figure 2 demonstrates these templates. The CoT approach frames the problem as a document classification task (for the presence or absence of one or more AIFs), with the intermediate steps being the generation of the sentences that would constitute AIFs. The ‘Standard' approach requests the AIFs only, and the classification label is determined by whether any AIFs are returned. We also experimented with formatting instructions for the outputs, JSON or free text. The AIFs extracted from reports would be stored and used in other applications, so a defined output format such as JSON is useful. However, there is evidence that constraining LLM outputs can be harmful to performance (32), so we experimented with both approaches. The prompts were preprocessed by using each LLM's tokenizer and instruction template.

### 2.4 QLoRA

Preliminary experiments demonstrated that in-context learning (33), a transfer learning technique where demonstration example(s) are provided in the prompt (e.g., ‘few-shot learning'), was not effective on this task. We instead utilised QLoRA, a technique that combines 4-bit model quantisation with LoRA (6, 8). The size of the models used in this project prohibits full fine-tuning (as would be standard with smaller language models such as BERT), and LoRA overcomes this by fine-tuning a subsection of the base model's weights. Early experiments revealed that model quantisation did not reduce model performance and provided the opportunity to trial larger models such as Phi-4. This would not have been feasible with our hardware without quantisation. The QLoRA approach reduced the amount of video random access memory (VRAM) required to fine-tune the LLMs to the task and made it feasible on a consumer-level GPU. Please see Supplementary material (Section 11) for the comparison table of 16-bit LoRA vs. (4-bit) QLoRA.

### 2.5 Inference/decoding

LLMs output probabilities for each token in the vocabulary at each generation timestep, and there are different strategies to convert these into text. Four such strategies were trialled: greedy sampling, nucleus sampling (34), beam search, and a hybrid approach combining greedy and nucleus sampling. Greedy sampling takes the most probable token at each timestep, whereas nucleus sampling probabilistically selects tokens. It introduces two parameters: Temperature which controls the amount of variability in the generations (35) and ‘top p’, which sets a probability threshold limiting the selection of tokens to those whose accumulated probabilities meet the threshold (34). Beam search generates multiple candidate answers (or ‘beams’) and then selects the candidate with the highest overall probability (36). We trialled four and eight beams. Once decoded, a rule-based parser removed artefacts from the text before verifying the generation is valid JSON. The hybrid decoding method combined greedy search and nucleus sampling. It worked as follows: If greedy search failed to result in valid JSON, nucleus sampling was attempted with both temperature and top *p* set to 0.5, only stopping when an attempt resulted in valid JSON, or a five-attempt limit was reached. In the latter case, a JSON-parsable ‘null’ answer would be returned and considered incorrect in evaluation. This ensures that even if the model cannot provide a valid answer, these errors are not propagated to downstream applications.

### 2.6 Hyperparameters

Optimal hyperparameters were found via experimentation on the validation dataset. The rank (‘r’) hyperparameter is particularly important as it contributes to how large the LoRA matrices are. We found setting *r* at 16 and alpha at 64 offered the best balance of performance and memory consumption. The models were trained for three epochs using a linearly decaying learning rate of 2 × 10^−4^ with an 8-bit AdamW optimiser (37). Eight gradient accumulation steps of mini-batch size of one were used (38), creating an overall batch size of eight.

### 2.7 Evaluation

To evaluate the model, we considered both document- and sentence-level performance. For document-level evaluation, we used accuracy, precision, recall, and F1 score metrics. As both the positive and negative classes at the document level are significant, we used the macro-average of precision, recall, and F1 score. A document-level positive label was defined as one or more sentences corresponding to AIF(s) being in the report, and a negative label was no AIF-related sentences being present.

An exact string match is the simplest method to compare the gold annotation sentences against the model's generations and guarantees semantic equivalence. This allows automatic sentence-level evaluation to provide the lowest estimate of performance (as an exact match guarantees the semantic accuracy of anything deemed correct). However, sentence boundaries can be ambiguous, and the model may determine them to be different from the sentence tokenizer (39). A common example of this was omitting the number at the start of a numbered list entry. This would be considered wrong as an exact string match but correct by an end user. Therefore, we normalised the sentences by removing whitespace, punctuation, and numbers from the beginning and end of generations and annotations. This alleviates the issue of evaluating correct incidentals as incorrect without jeopardising the meaning of the sentence. Precision, recall, and F1 score were used to evaluate the sentences once normalised with no macro-averaging. We also qualitatively analysed errors to account for examples that our normalisation process does not account for, as these would be marked as incorrect even if semantically equivalent, and assessed other generation characteristics which may affect how the system would perform in practice.

Neural network training involves a degree of randomness, so the final models used for external evaluation were trained three times with three random seeds before any evaluation on the internal or external test sets took place. This allowed the mean and 95% confidence intervals for the quantitative metrics outlined above to be reported, while avoiding any test set bias during development.

The final evaluation consideration was whether the LLM always generates parsable output or produces errors. These errors were recorded when comparing different decoding and prompting techniques for further comparison.

## 3 Results

Our best performing model, a QLoRA-adapted Llama-3.1-8B Instruct with the CoT-JSON prompting strategy, achieved strong performance on internal data and demonstrated generalisability to external data.

**Figure 3:**
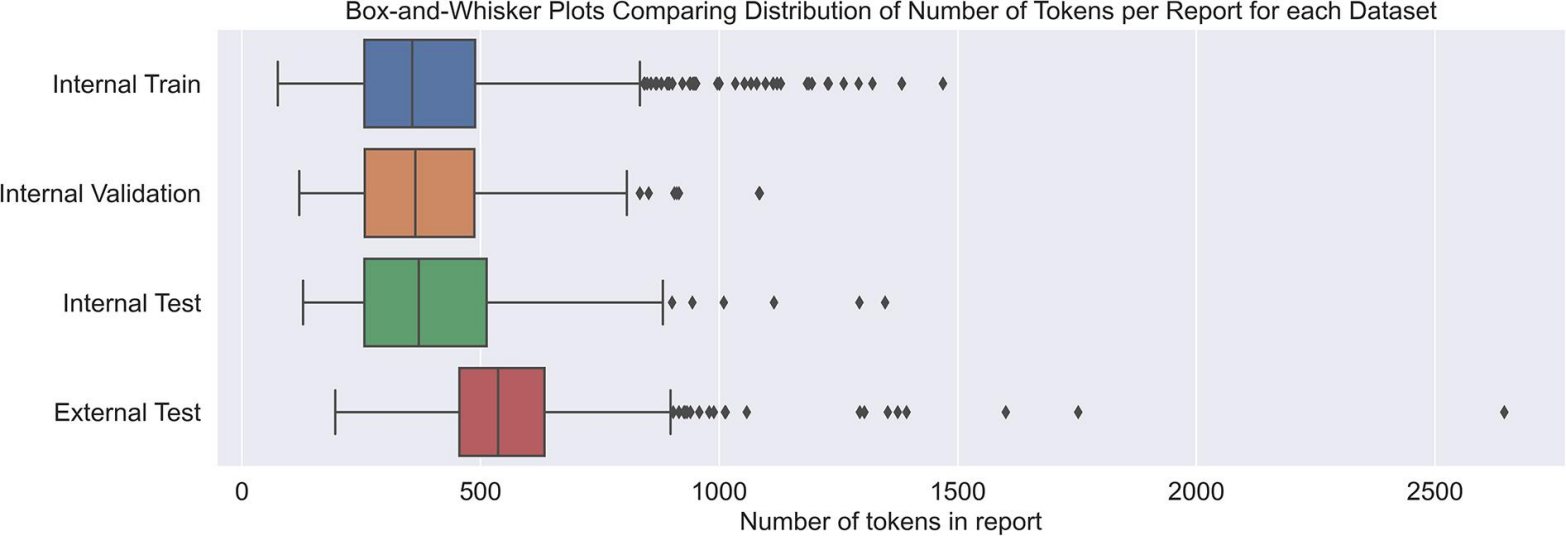
Box-and-whisker plot demonstrating the difference in token lengths of reports between internal and external reports. Token counts were using the Llama 3.1 8B Instruct tokenizer.

In terms of dataset characteristics, the inter-annotator agreement measured 0.75 using Cohen's kappa, signifying either ‘substantial’ or ‘excellent’ agreement (40, 41), before the disagreements were resolved. Table 1 outlines quantitative differences between the internal and external datasets. The external reports were noticeably longer and contained more AIF sentences per report. The class distribution at the document level was also different, with most external reports being positive compared with a minority of internal reports. Figure 3 shows how the median length of external reports was both greater and lay outside the interquartile range of the internal datasets. External outliers could also be much longer than the internal outliers.

**Table 1:** Statistics of the datasets used in this study.

Dataset	No. of reports	No. of patients	AIF positive reports	AIF negative reports	AIF positive/negative ratio	Mean tokens per report	Mean AIF sentences per report
Internal training	1,999	1,847	786	1,213	0.64	392	0.781
Internal validation	248	230	103	145	0.71	391.9	0.806
Internal test	250	231	104	146	0.71	405.9	0.908
External test	460	N/A	286	174	1.64	570.3	1.697

Mean tokens per report is derived using Llama 3.1 8B's tokenizer. Individual patient information was not available for the external test set.

**Figure 4:**
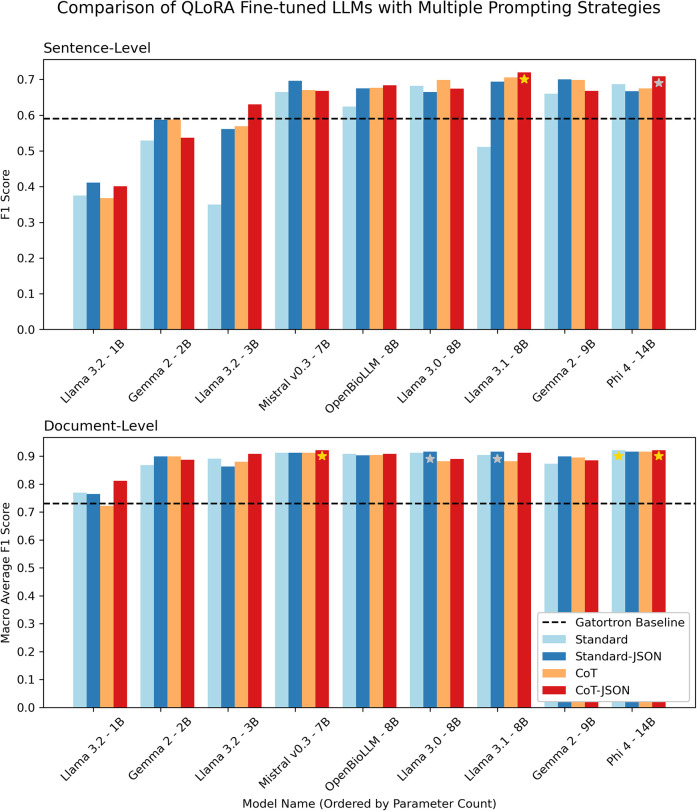
Grouped bar chart demonstrating sentence-level F1 scores and document-level macro-average F1 scores on the validation dataset for LLMs with parameter counts ranging from 1 to 14 billion parameters using four different prompting strategies. Gold and silver stars represent the first and second highest F1 scores at the sentence and document level. The dashed black line shows the performance of the baseline encoder-only GatorTron model (fine-tuned as a binary sentence classifier).

Figure 4 demonstrates document-level macro-average F1 scores and sentence-level F1 scores for different fine-tuned LLMs on the validation set when using the four different prompt strategies outlined in Figure 2. The document-level classification was close across all models and prompt strategies, with only Llama 3.2-1B (the smallest LLM tested) achieving an F1 score below the GatorTron baseline. The sentence-level component of the task was where the larger models performed significantly better. The best performing models were Llama 3.1 8B and Phi-4 14B using the CoT-JSON prompt strategy. Llama 3.1 8B was ultimately chosen for further internal and external evaluation as it had a marginally higher mean of document and sentence-level metrics (0.816 vs. 0.815 for Phi-4) and achieved this performance with 57% of the parameters of Phi-4, making it significantly more efficient.

Table 2 shows the performance of the Llama 3.1 8B model on both test datasets with different decoding methods. Strong performance was observed on the internal data using the automatic evaluation approach, but we see a drop in all document-level and sentence-level metrics on the external data. On internal data, hybrid, greedy, and nucleus sampling performed similarly; however, on the external data, the greedy and hybrid decoding methods performed the best. Hybrid decoding scored higher due to the greedy method failing to output JSON on one example. The hybrid method overcame this with its nucleus sampling fallback. Looking at the beam search performance, eight beams outperformed four beams but fell short of the hybrid, greedy, and nucleus sampling techniques on both datasets.

**Table 2:** Comparison of decoding strategies using QLoRA fine-tuned Llama 3.1 8B Instruct model on both the internal and external test datasets.

Dataset	Decoding strategy	Document-level (macro-average)				Sentence-level			Parsing errors
		Precision	Recall	F1	Accuracy	Precision	Recall	F1	
Internal test	Hybrid	0.919 ± 0.017	**0.916** **± 0.016**	**0.917** **± 0.016**	**0.92** **± 0.016**	0.787 ± 0.009	**0.724** **± 0.013**	0.754 ± 0.011	**0**
	Greedy	0.919 ± 0.017	**0.916** **± 0.016**	**0.917** **± 0.016**	**0.92** **± 0.016**	0.787 ± 0.009	**0.724** **± 0.013**	0.754 ± 0.011	**0**
	Nucleus	**0.92** **± 0.008**	0.915 ± 0.008	**0.917** **± 0.008**	**0.92** **± 0.008**	0.79 ± 0.025	**0.724** **± 0.016**	**0.756** **± 0.02**	**0**
	Beam (4 beams)	0.891 ± 0.004	0.879 ± 0.014	0.883 ± 0.011	0.888 ± 0.009	0.805 ± 0.032	0.607 ± 0.04	0.691 ± 0.016	**0**
	Beam (8 beams)	0.897 ± 0.004	0.889 ± 0.004	0.892 ± 0.001	0.896 ± 0.0	**0.816** **± 0.034**	0.649 ± 0.045	0.722 ± 0.032	**0**
External test	Hybrid	0.797 ± 0.019	0.815 ± 0.02	**0.79** **± 0.025**	**0.793** **± 0.025**	0.588 ± 0.021	**0.47** **± 0.024**	**0.522** **± 0.012**	0–1
	Greedy	0.796 ± 0.018	0.815 ± 0.02	**0.79** **± 0.024**	0.792 ± 0.024	0.589 ± 0.018	0.468 ± 0.022	0.521 ± 0.012	0–5*
	Nucleus	**0.798** **± 0.019**	**0.816** **± 0.02**	0.789 ± 0.024	0.791 ± 0.025	0.585 ± 0.023	0.463 ± 0.019	0.517 ± 0.01	**0**
	Beam (4 beams)	0.773 ± 0.011	0.786 ± 0.008	0.754 ± 0.009	0.755 ± 0.01	**0.7** **± 0.027**	0.392 ± 0.018	0.503 ± 0.018	**0**
	Beam (8 beams)	0.787 ± 0.009	0.803 ± 0.007	0.773 ± 0.005	0.775 ± 0.005	0.696 ± 0.026	0.405 ± 0.007	0.512 ± 0.012	**0**

The best performing metrics on each dataset are highlighted in bold. A parsing error occurs when the model has to retry the generation. Each model was trained three times, with three different random seeds, and we report the mean and 95% confidence intervals for each metric, along with the range of parsing errors (per run) over three runs.

* Represents that this parsing error was fatal, and after five attempts, a ‘null’ response was provided and automatically marked as incorrect.

Table 3 shows the effect of the different prompt templates (Figure 2) on performance on both internal and external data (using hybrid decoding). The prompt combining both CoT and JSON output instructions provided the best document-level performance on both datasets and the best overall sentence-level F1 score on the external data, primarily due to its better recall. Due to using different parsers for JSON and non-JSON outputs, the parsing error scores serve as a point of comparison only within the same output format. In this regard, the CoT-JSON prompt outputs were also more robust to JSON parsing errors on the external data than the standard prompting approach.

**Table 3:** Comparison of prompt strategies using Llama 3.1 8B Instruct on the internal and external test datasets.

Dataset	Prompt strategy	Document-level (macro-average)				Sentence-level			Parsing errors
		Precision	Recall	F1	Accuracy	Precision	Recall	F1	
Internal test	CoT—JSON	**0.919** **± 0.017**	**0.916** **± 0.016**	**0.917** **± 0.016**	**0.92** **± 0.016**	0.787 ± 0.009	**0.724** **± 0.013**	0.754 ± 0.011	**0**
	Standard—JSON	0.891 ± 0.041	0.88 ± 0.045	0.885 ± 0.043	0.889 ± 0.041	**0.82** **± 0.049**	0.706 ± 0.016	**0.758** **± 0.013**	**0**
	CoT	0.894 ± 0.005	0.882 ± 0.005	0.887 ± 0.005	0.892 ± 0.005	0.71 ± 0.152	0.68 ± 0.025	0.69 ± 0.072	**0** *
	Standard	0.902 ± 0.004	0.897 ± 0.008	0.899 ± 0.006	0.903 ± 0.005	0.647 ± 0.124	0.705 ± 0.031	0.67 ± 0.049	**0** *
External test	CoT—JSON	**0.797** **± 0.019**	**0.815** **± 0.02**	**0.79** **± 0.025**	**0.793** **± 0.025**	**0.588** **± 0.021**	**0.47** **± 0.024**	**0.522** **± 0.012**	**0–1**
	Standard—JSON	0.785 ± 0.015	0.801 ± 0.015	0.775 ± 0.008	0.778 ± 0.008	0.579 ± 0.022	0.459 ± 0.025	0.512 ± 0.01	3–4
	CoT	0.793 ± 0.005	0.81 ± 0.006	0.782 ± 0.007	0.784 ± 0.008	0.544 ± 0.086	0.466 ± 0.011	0.501 ± 0.041	**0** *
	Standard	0.793 ± 0.014	0.811 ± 0.014	0.784 ± 0.013	0.787 ± 0.012	0.349 ± 0.08	0.455 ± 0.012	0.393 ± 0.054	**0** *

Best performing metrics on each dataset are marked in bold. Each model was trained three times, with three different random seeds, and we report the mean and 95% confidence intervals for each metric, along with the range of parsing errors over three runs.

* Denotes metric derived using a whitespace parser rather than the more stringent JSON parser, so * values should not be compared with ‘non *’ values in the ‘Parsing Errors’ column.

**Figure 5:**
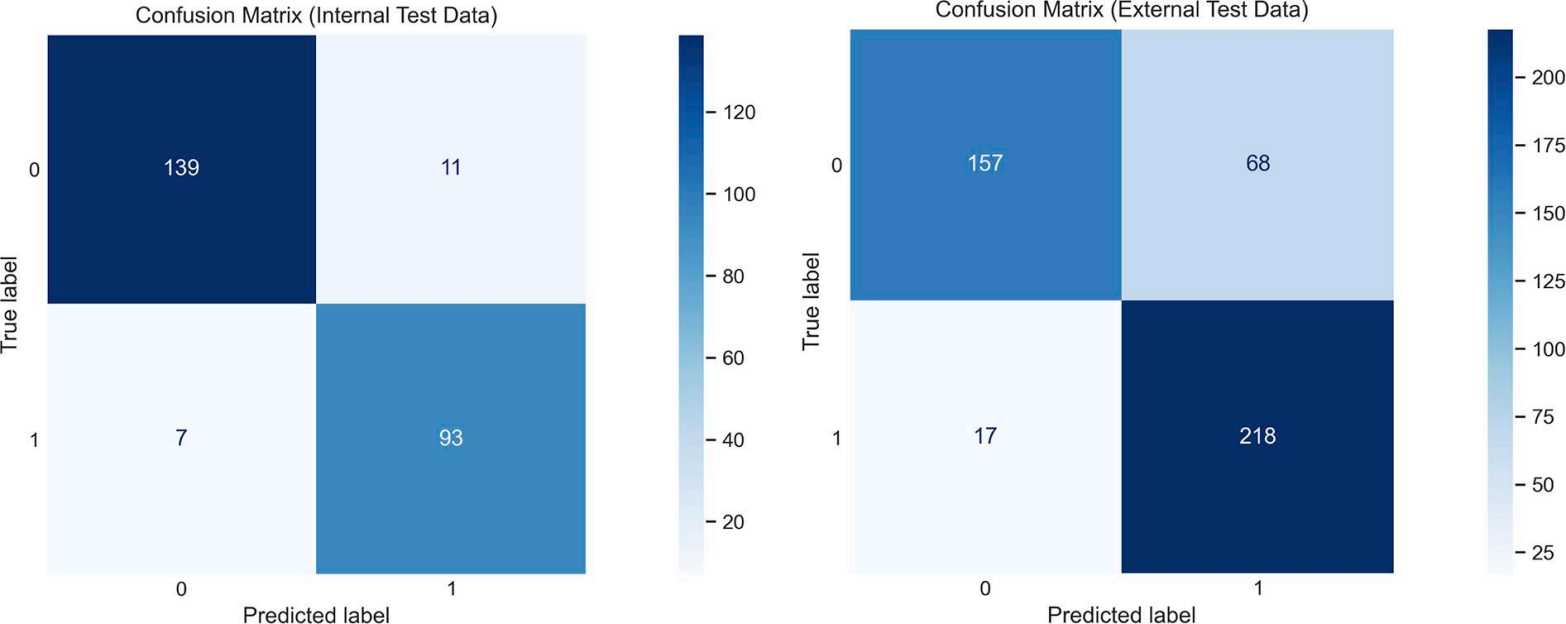
Confusion matrices demonstrating the document-level classifications on the internal and external datasets, using the final selected Llama 3.1 8B Instruct model trained with the CoT prompt with JSON output, and using the hybrid decoding method.

Figure 5 displays confusion matrices of the document-level classification for both test datasets. The performance on the internal data was strong, with the errors being split evenly between false positives and false negatives. With the external data, there was an increase in false positives that resulted in lower classification performance.

**Figure 6:**
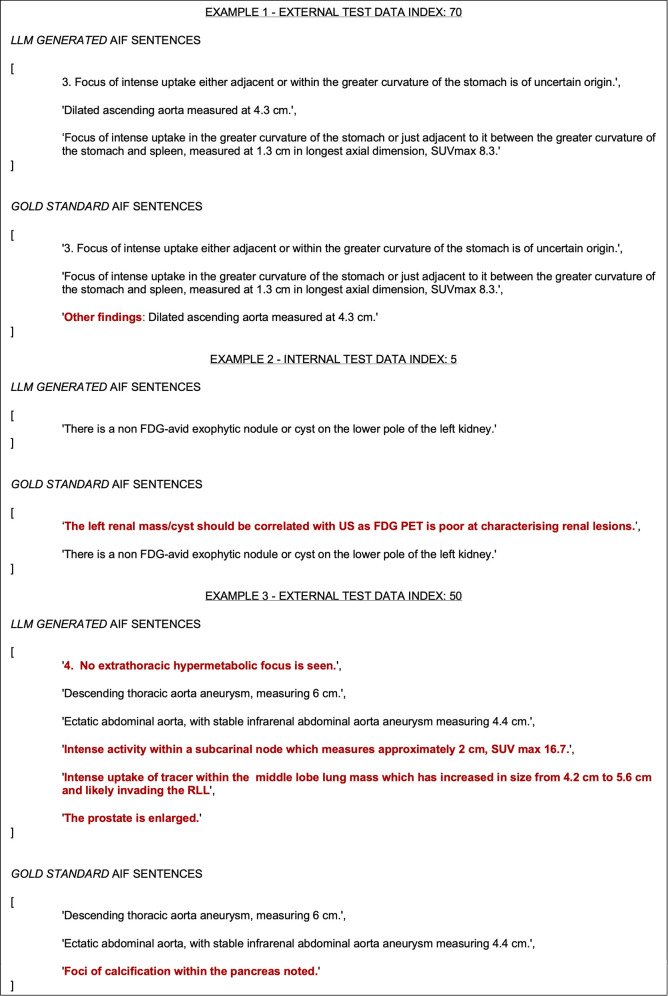
Three error analysis examples from the internal and external test sets. The difference between the gold standard sentences and the model-generated sentences is highlighted in red.

Figure 6 shows generations from the model compared against the gold standard data. Errors can be described as ‘soft' or ‘hard’ depending on whether they would realistically affect clinical practice. Three key soft error types were identified: (1) ‘sentence boundary errors’, (2) ‘multiple-reference errors’, and (3) ‘text artefact errors’. The automatic evaluation process would classify these as incorrect, but they are correct in practice (or at least unharmful). Hard error types included (1) ‘contextual misinterpretation of actionability’, (2) ‘missed AIF finding’, and (3) ‘not an AIF’. These are also evaluated as incorrect by the automatic evaluation process and are true mistakes. Some of the examples in Figure 6 reveal examples where the automatic evaluation metrics penalised the model for soft errors. The first example shows how the model generated the correct AIF sentences from the report, but did not include ‘other findings’: at the start of one AIF, a ‘sentence boundary error'. This error resulted in both a false positive and false negative being recorded, even though the model is semantically correct. This error occurs due to an annotation decision, where it could be argued that either sentence boundary is appropriate. Example 2 shows how the model generates a reference to the only AIF in the report, a nodule on the left kidney, but misses the other reference to the same AIF (a ‘multiple-reference error’). This is unlikely to make a difference in clinical practice. Example 3 demonstrates some true (‘hard’) errors, all accounted for correctly by the automatic evaluation. The false positive enlarged prostate finding is worth noting as this was in the original report and is an incidental finding, but not considered ‘actionable’ by the annotators (a ‘contextual misinterpretation of actionability’ error).

## 4 Discussion

We developed a deep learning pipeline utilising open LLMs that classifies FDG PET-CT radiology reports for the presence or absence of AIFs by also extracting the key sentences that refer to them. Our quantitative evaluation approach demonstrates an impressive lowest estimate of performance on the internal data, with a document-level macro-average F1 score of 0.925 and a sentence-level F1 score of 0.745. On external data, this lowest estimate of performance drops to 0.812 and 0.524, respectively, demonstrating domain shift between the two hospitals reporting, which causes difficulty for LLMs. However, error analysis demonstrated that the ‘real-world’ performance is higher on both datasets, with correct answers being penalised due to complications surrounding sentence boundaries and writing styles. The model can label and extract sentences from thousands of reports in a fraction of the time it would take an expert. Accordingly, this pipeline has the potential to be used for both real-time clinical alerts and reminders when reports are submitted and retrospective analysis of comorbidities from past reports.

The various LLMs performed differently on the document- and sentence-level parts of the task (Figure 4). Interestingly, most models perform well at a document-level, but sentence-level performance generally increases with the number of parameters in the base LLM. For the sentence-level component, the model must distinguish between sentences referring to lung cancer and unexpected incidental findings and then, within these sentences, distinguish between actionable and non-actionable findings. This is a complex task that all but highly trained experts would find challenging. It follows that more parameters in a model would increase its ability to make these distinctions. Llama 3.1 8B outperforming Phi-4 14B demonstrates that raw parameter count is not the only factor, however.

The results also demonstrate how choosing the correct prompting technique for a given LLM is important for the more challenging sentence-level task (Figure 4). For example, Llama 3.1 8B was the best performing with a ‘CoT-JSON’ strategy but performed poorly with the ‘Standard' prompt. In contrast, the Mistral model tested was more invariant to prompt changes but slightly worse overall. The middling performance of OpenBioLLM also suggested that further medical domain adaptation seems less important with larger models, when compared with the more significant benefits with smaller models reported in previous work (16). The significant increase in the number of tokens these models are trained on (∼90 billion tokens for GatorTron, ∼15 trillion tokens for Llama 3.1) may minimise the advantage of further specialisation (23, 30).

The challenge of evaluating generative models is widely discussed in the field (42–44). The specific nature of our single-task system (as opposed to a general chatbot/assistant, etc.) allowed us to approach evaluation differently from other LLM projects. We utilised a two-stage approach, firstly using a quantitative *guaranteed* assessment of the worst-case performance of the model and secondly a qualitative approach using actual examples from the model. The error analysis in this second phase revealed many examples where the quantitative approach punished the model unfairly. Despite these strengths in evaluation, our results suggest some potential implementation challenges. The increase in false positives in Figure 5 could cause ‘alert fatigue’ in an end user (a concern in the wider field) (45, 46). Future work would be to develop user guidance in consultation with nuclear medicine physicians to ensure it helps and does not hinder their practice. We would also like to develop a robust human evaluation protocol for the model, as this could inform how it is inserted into clinical workflows, and how to best utilise the extracted AIFs in downstream practice. However, human evaluation of LLMs is labour-intensive and has its own shortcomings to be overcome (47, 48).

This study demonstrated some interesting findings regarding LLM generalisation. The two datasets have different characteristics (Table 1 and Figure 4), and previous work with this dataset noted that the internal and external reports used a different reporting style (16). The internal reports order the findings section by *priority* where the most significant findings are stated first, whereas the external reports order findings *anatomically* (i.e., sequentially from head down to legs). Rohren (49) provides details on these established styles. Table 1 and Figure 4 show that an anatomical reporting style potentially results in significantly longer reports with more AIF sentences. There is evidence that longer reports are harder to understand for humans, and perhaps LLMs find longer reports more challenging also (50). This difference in performance could also have implications in deployment if a hospital changes its imaging reporting protocols.

LLMs can hallucinate, where they generate incorrect but seemingly plausible content (51), an issue of concern in the healthcare domain (52). Throughout our evaluation, we found no instances of hallucination: All extracted sentences, correct or not, were present in the original report text. This reliability is likely because we have adapted the LLM for a single task, and this has successfully enforced consistency on new examples. This provides confidence in the real-world deployment of such a system. A caveat is that we were unable to formally check every individual generation for hallucinations.

There is increasing evidence that LLMs perform better when trained with explicit reasoning steps, most notably with DeepSeek-R1 (53). We found that even a simple CoT prompting approach results in better document-level performance on both test datasets (Table 3). We also found that training the LLM to output JSON generally improved performance in contrast with previous work (32). LLM prompt engineering is widely discussed when using closed LLMs such as ChatGPT for inference (54), but less so when fine-tuning models for specific tasks. We found that it makes a performance difference and argue that it always needs investigation when developing an LLM-based system.

In terms of decoding, we found that the computationally cheaper greedy and nucleus searches worked better than beam search (Table 2). This is likely due to how these decoding styles are more aligned with the next-token prediction objective. The hybrid approach proposed improved on these decoding styles, allowing the model to escape JSON parsing errors by using nucleus search as a fallback for greedy decoding when it failed to provide parsable output. The hybrid approach also ensures that a JSON-parsable ‘null’ error is returned in the event of a challenging report that the model cannot solve, ensuring downstream applications are not affected. Another advantage of non-beam approaches is an increase in the speed of processing and less memory required for processing.

Our approach only requires one consumer-level GPU, not only making it more accessible from a resources standpoint but also demonstrating that LLM benefits can be harnessed at a lower energy cost than an application such as ChatGPT would at inference time (55).

A key limitation of the model is that it is designed for lung cancer, and due to the inherently contextual nature of incidental findings, it cannot be guaranteed to work for other conditions. We believe the AIF extraction methodology presented could be applied to other imaging modalities and conditions, however. Two possible extensions we are looking at addressing in future work are prostate cancer scans for PSMA (prostate-specific membrane antigen) PET-CT and AIFs in brain MRI scans (56). The extensible nature of LoRA adapters means a future ‘mixture-of-experts’ model for AIFs could utilise all these models as part of one comprehensive system (57). This work would serve as a blueprint for training the ‘experts’ in such a system. Another limitation is the number of data points and annotators available for the project, an issue for most supervised learning tasks. Limitations on expert annotator time also prevented us from testing inter-annotator agreement on the internal data. This could result in some bias in the trained model towards the sole annotator's judgement; however, since the agreement of the two annotators was consistent on external data, and the internal data are from the same hospital as both annotators, we considered this a reasonable compromise and the best use of the expert annotation hours available. Especially considering agreement was still quantified on the external data. We were also unable to stratify performance based on different demographic groups, which would in turn have allowed us to evaluate the fairness and bias of the model. The ethical approval and data retrieval process for the initial study, this dataset was used in ensured anonymity to an extent that the relevant features were not in our dataset (16). We acknowledge this is an important step for acceptance of such models and hope to incorporate these experiments into future work. Finally, although we use open LLMs in this work to ensure repeatability, these models cannot be considered truly open source. For example, our final model utilised Llama 3.1 8B Instruct, which has open weights, but the exact pretraining data makeup was not published (23).

## 5 Conclusion

We developed an LLM-based model that both classifies PET-CT reports for the presence or absence of AIFs and extracts the sentences from the text that inform that classification. We demonstrate its efficacy by quantitatively and qualitatively analysing its performance on both internal and external reports. We believe this model would be effective in assisting clinicians by providing real-time alerts and reminders and for future analysis of AIFs in patient histories.

## Data Availability

The raw data supporting the conclusions of this article will be made available by the authors, without undue reservation.
