# Supplementary material for: Open LLM-based actionable incidental finding extraction from [18F]fluorodeoxyglucose PET-CT radiology reports
Source: Front Digit Health. 2025 Nov 14;7:1702082. doi: 10.3389/fdgth.2025.1702082 (PMC12660291; doi:10.3389/fdgth.2025.1702082)
Supplement: Supplementary file 1 [file Datasheet1.pdf]

Supplementary Material

1 Hyperparameter Tables

1.1 Training Hyperparameters

	Values Trialled (Bold Signifies Chosen Value)									
Max Sequence Length	2048	**4096**	8192							
Lora Rank (r)	1	2	4	8	**16**	32	64	128	256	512
Lora Alpha (α)	2	4	8	16	32	**64**	128	256		
Lora Dropout	**0**									
Starting Learning Rate	9.00E-05	1.00E-04	**2.00E-04**	3.00E-04						
Mini Batch Size	**1**	2								
Gradient Accumulation Steps	4	**8**	16							
Epochs	1	2	**3**	4	5					
Warmup Steps	0	5	**10**	20						

Supplementary Table 1. List of training hyperparameters and the values trialled during preliminary experiments. Final values chosen are highlighted in bold.

1.2 Generation Hyperparameters

	Values Trialled (Bold Signifies Chosen Value)					
Strategy	Greedy	Sampling	Beam Search	**Hybrid**		
Temperature (Sampling)	0.1	0.3	**0.5**	0.7	0.9	1
Top p (Sampling)	0.1	0.3	**0.5**	0.7	0.9	1
Number of Beams (Beam)	4	8				

Supplementary Table 2. List of generation/decoding hyperparameters and values trialled during preliminary experiments. Final values chosen are highlighted in bold.

2 Sentence Level Extraction

AIF extraction at sentence-level could be considered a specific case of extractive summarization (1) where actual passages from the source text are used to create a shorter version containing all salient points. This is usually evaluated using the metrics BLEU and ROUGE (2, 3).These methods both measure n-gram (a sequence of n length symbols, in this case 'words') overlap between a candidate summary and gold standard summary, with BLEU prioritising precision, and ROUGE recall. However this character level overlap provides no guarantee of *semantic* similarity, and their suitability as metrics has been questioned (4, 5). We determined this makes them unsuitable for this project, where subtle changes in the way a finding is phrased could make an outsized difference clinically. This task differs from extractive summarization in that we are not concerned with the order of the sentences, and only a narrow subset of the total sentences in a report are required, if any (i.e. a report with no AIFs needs no AIF summary). This allowed us to take a different approach to evaluation, namely using exact string matches.

3 Metric Equations

TP = True Positives, TN = True Negatives, FP = False Positives, FN = False Negatives
n = Number of possible classes e.g. for binary classification $n = 2$.

$$Accuracy = \frac{TP + TN}{TP + TN + FP + FN}$$

$$Precision\ (or\ PPV) = \frac{TP}{TP + FP}$$

$$Recall\ (or\ Sensitivity) = \frac{TP}{TP + FN}$$

$$F1\ Score = 2 \times \frac{Precision \times Recall}{Precision + Recall}$$

$$Macro\ average\ Precision = \frac{\sum_{i=1}^{n} Precision_i}{n}$$

$$Macro\ average\ Recall = \frac{\sum_{i=1}^{n} Recall_i}{n}$$

$$Macro\ average\ F1\ Score = \frac{\sum_{i=1}^{n} F1\ Score_i}{n}$$

Please note we do not use AUROC (area under receiver operating curve) in this work as the generative model does not provide the conventional document-level sigmoid/softmax probability scores needed to create an informative receiver operating curve, and the resulting area would therefore be ambiguous.

4 Further Annotation Guideline Examples and Guidance

4.1 High Level Guidelines

Actionable findings as defined and explained in both American College of Radiology (ACR) and Royal College of Radiologists (RCR) white papers if not related to the existing lung cancer are included as AIFs. Links to these documents are below.
https://www.acr.org/Clinical-Resources/Clinical-Tools-and-Reference/Incidental-Findings
https://www.rcr.ac.uk/our-services/all-our-publications/clinical-radiology-publications/recommendations-on-alerts-and-notification-of-imaging-reports/
Further guidance in the RCR pamphlet *Management of Incidental Findings Detected During Research Imaging* (REF), although primarily relating to research subjects as opposed to typical patients, provided further UK-centred guidance in defining what incidental findings should result in clinical 'action' (and therefore be included in our annotation definition).
https://www.rcr.ac.uk/our-services/all-our-publications/clinical-radiology-publications/management-of-incidental-findings-detected-during-research-imaging/
These sources were adapted for our specific use case to define an AIF in this project as "any finding that would require medical intervention ("action") for a lung cancer patient". These must be distinct from known or suspected lung cancer. These are findings that a PET-CT nuclear medicine consultant would expect the referring physician to act upon.

4.2 Examples of Specific Borderline Distinctions (Not Exhaustive)

Include: Any emphysematous finding with an 'intensifying' qualifier. E.g "Severe", "extensive", "widespread.
Exclude: "Mild", "Moderate", Emphysema (or just "Emphysema on its own" etc…).
Include: Abdominal aortic aneurysms measuring 3cm or greater.
Exclude: Dilated aorta below 3cm. (This guideline is based on the recommendations from this review paper: https://www.sciencedirect.com/science/article/abs/pii/S1050173819301562)
Include: Other neoplastic findings that are not related to the lung cancer. E.g. an incidental prostate cancer.
Exclude: Developments or consequences of the known or suspected lung cancer. E.g. secondary primaries, nodal involvement, metastasis.
Include: Fractures unrelated to lung cancer. E.g. Wedge compression fractures in the spine from osteoporosis or trauma.
Exclude: Pathological fractures which are the result of lung cancer metastasis.

4.3 Technical Guidelines

PET-CT reports are typically composed of two sections (usually "Findings" and "Interpretation"). Annotate the first sentence referring to an AIF in each section of the report, but do not label any further sentences unless they themselves introduce a new AIF. The rationale for this is as follows: The number of times a finding is mentioned is significant as an indication of severity, but brevity is important in having a succinct reference to the finding with a clinician being able to search for more detail if necessary.

In cases where a sentence contains more than one AIF, (e.g. "We note fatty liver and a 4.5cm AAA") this is annotated as one AIF sentence.
Any finding where the original reporter recommends an intervention is considered an AIF. For example, a sentence like "Increased uptake in rectum should be correlated with ultrasound" is always considered an AIF.

Supplementary material is not typeset so please ensure that all information is clearly presented, the appropriate caption is included in the file and not in the manuscript, and that the style conforms to the rest of the article.

5 System Flow Chart

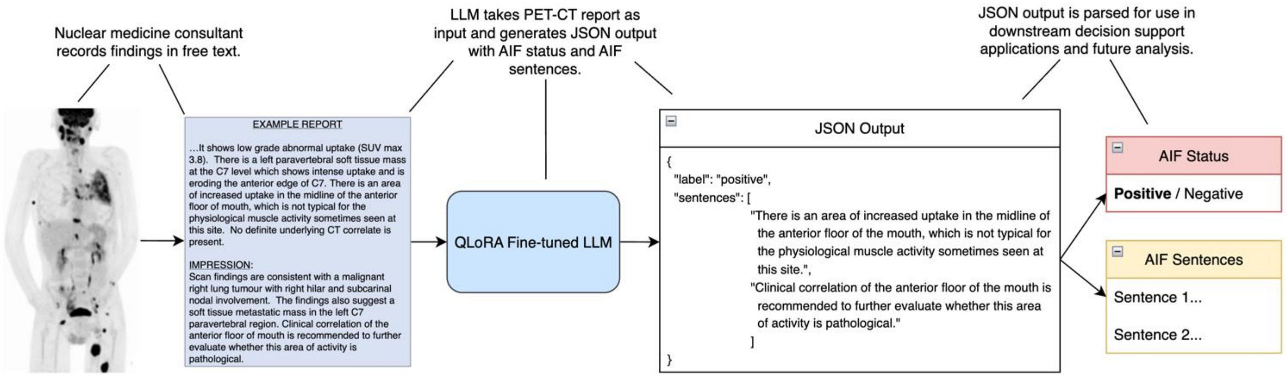

Supplementary Figure 1. Flow chart demonstrating the proposed system in use.

6 Pre-processing: Modified Sentence Tokenizer

The reports were split into sentences using a modified NLTK tokenizer to differentiate between AIF and non-AIF sentences (6). This adapted the NLTK sentence tokenizer with a rule-based component to better suit radiological purposes. The entire report was inputted to the model as part of the prompt. The main adaptation in our sentence tokenizer was a rule being keeping numbered list entries as one sentence. For example, the sentence "2. No evidence of metastatic disease." should be kept intact rather than split into "2." and "No evidence of metastatic disease.", which we found would always happen with the default sentence tokenizer. For consistency and replicability, where the human annotated sentences differed from the equivalent tokenizer derived sentence, we would use the automatic version as the final labels (unless it was grammatically incorrect). It should be noted that the sentence tokenization only affected the labelling and the evaluation. The reports were fed to the LLM as part of the prompt in their entirety.

7 Additional Note on LLMs

Autoregressive language models are pre-trained on large amounts of data with the objective of predicting the next token (a token in this case being somewhere between a syllable and a word, to distinguish from the sentence splitting mentioned earlier). This allows the model to learn both complex linguistic patterns from the training corpus, and the ability to generate coherent new text. Most open LLMs use a decoder-only transformer architecture with many trainable parameters (typically between 1 and 405 billion parameters for public models) (7-9).

8 Explanation of QLoRA

LoRA finetuning is a process where a (relatively) small set of parameters are trained while the initial LLM remains fixed. These sets are often referred to as adapters and have another benefit of being able to either be stored separately or merged with the initial LLM. This project looked at one task, but it is possible to train multiple adapters for different tasks which are compatible with the same base LLM. We envision this benefit could be very useful for tailoring LLMs for specific clinical tasks that the base model performs poorly on. QLoRA builds on LoRA by using two different data types in the fine-tuning process, a "storage" 4-bit data type (NormalFloat4) and a 16-bit "computation" data type (BrainFloat 16-bit) (10). This dramatically reduces the amount of memory required to store both the LLM, and the backpropagated gradients created, when fine-tuning the LoRA adapter. Further memory and speed optimizations were utilised using the Unsloth software library (11). The figure below demonstrates QLoRA diagrammatically.

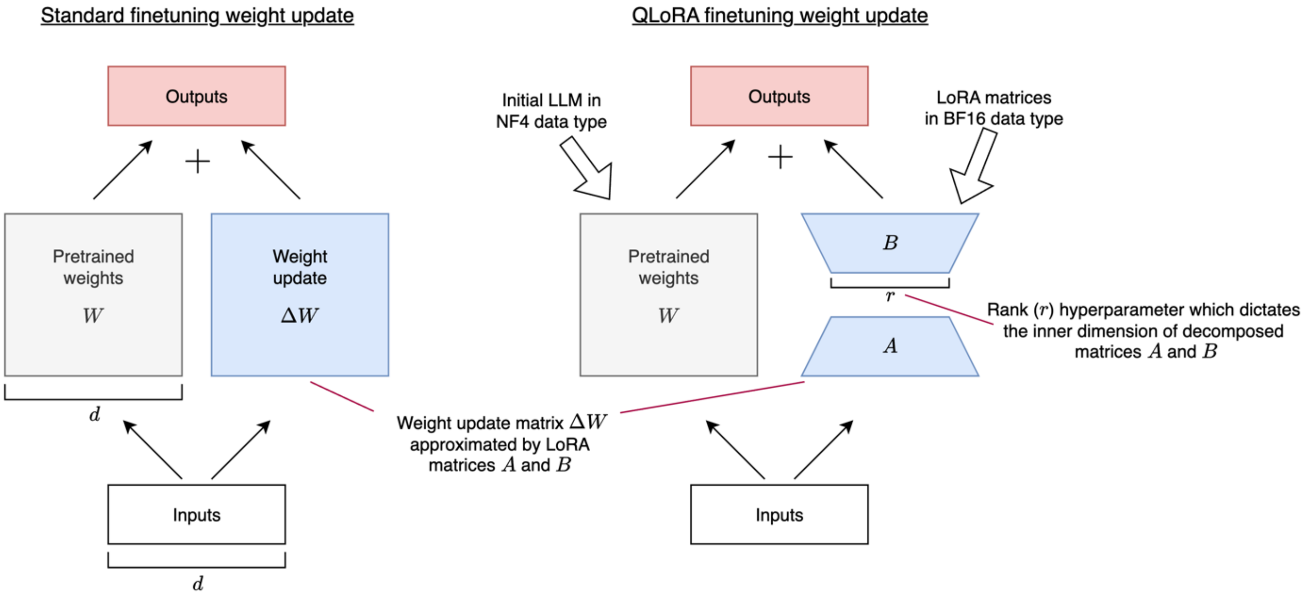

Supplementary Figure 2. Diagram demonstrating how QLoRA weight updates approximate a traditional 'standard' fine-tuning step by updating a subset of the total weights of the model.

9 Supplementary Results Tables

Base Model	Prompt Strategy	Document Level				Sentence Level		
		Precision	Recall	F1	Accuracy	Precision	Recall	F1
GatorTron Baseline	N/a	0.8	0.77	0.73	0.73	0.443	**0.885**	0.59
Llama 3.2 1b	CoT - JSON	0.828	0.805	0.812	0.823	0.453	0.36	0.401
	CoT	0.727	0.719	0.722	0.734	0.34	0.4	0.368
	Standard - JSON	0.778	0.76	0.765	0.778	0.407	0.415	0.411
	Standard	0.811	0.762	0.77	0.79	0.442	0.325	0.375
Gemma 2 2b	CoT - JSON	0.889	0.886	0.887	0.891	0.55	0.525	0.537
	CoT	0.906	0.895	0.899	0.903	0.591	0.585	0.588
	Standard - JSON	0.908	0.893	0.899	0.903	0.584	0.59	0.587
	Standard	0.885	0.859	0.868	0.875	0.562	0.5	0.529
Llama 3.2 3b	CoT - JSON	0.911	0.906	0.908	0.911	0.658	0.605	0.63
	CoT	0.9	0.871	0.88	0.887	0.558	0.58	0.569
	Standard - JSON	0.882	0.855	0.863	0.871	0.596	0.53	0.561
	Standard	0.897	0.886	0.891	0.895	0.244	0.62	0.35
Mistral 7b v0.3	CoT - JSON	**0.923**	**0.919**	**0.921**	**0.923**	0.657	0.68	0.668
	CoT	0.919	0.906	0.912	0.915	0.697	0.645	0.67
	Standard - JSON	0.917	0.908	0.912	0.915	0.724	0.67	0.696
	Standard	0.917	0.908	0.912	0.915	0.692	0.64	0.665
OpenBioLLM 8b	CoT - JSON	0.911	0.906	0.908	0.911	0.692	0.675	0.684
	CoT	0.906	0.902	0.904	0.907	0.689	0.665	0.677
	Standard - JSON	0.909	0.9	0.903	0.907	0.691	0.66	0.675
	Standard	0.911	0.906	0.908	0.911	0.644	0.605	0.624
Llama 3.0 8b	CoT - JSON	0.901	0.884	0.89	0.895	0.711	0.64	0.674
	CoT	0.889	0.878	0.882	0.887	0.72	0.68	0.699
	Standard - JSON	0.921	0.913	0.916	0.919	0.692	0.64	0.665
	Standard	0.914	0.911	0.912	0.915	0.683	0.68	0.682
Llama 3.1 8b	CoT - JSON	0.914	0.911	0.912	0.915	0.72	0.72	**0.72**
	CoT	0.889	0.878	0.882	0.887	0.723	0.69	0.706
	Standard - JSON	0.919	0.914	0.916	0.919	0.703	0.685	0.694
	Standard	0.906	0.902	0.904	0.907	0.421	0.65	0.511
Gemma 2 9b	CoT - JSON	0.9	0.877	0.885	0.891	0.718	0.625	0.668
	CoT	0.902	0.89	0.895	0.899	0.72	0.68	0.699
	Standard - JSON	0.908	0.893	0.899	0.903	**0.732**	0.67	0.7
	Standard	0.884	0.867	0.873	0.879	0.67	0.65	0.66
Phi-4 14b	CoT - JSON	**0.923**	**0.919**	**0.921**	**0.923**	0.699	0.72	0.709
	CoT	0.919	0.914	0.916	0.919	0.686	0.665	0.675
	Standard - JSON	**0.923**	0.911	0.916	0.919	0.714	0.625	0.667
	Standard	**0.923**	**0.919**	**0.921**	**0.923**	0.683	0.69	0.687

	Prompt Strategy	Seed	Document Level Precision	Recall	F1	Accuracy	Sentence Level Precision	Recall	F1	Parsing Errors
Internal Test	CoT - JSON	1	0.928	0.923	0.925	0.928	0.779	0.714	0.745	0
		2	0.927	0.925	0.926	0.928	0.788	0.722	0.754	0
		3	0.902	0.9	0.901	0.904	0.795	0.736	0.764	0
		Mean ± 95% CI	**0.919±0.017**	**0.916±0.016**	**0.917±0.016**	**0.92±0.016**	0.787±0.009	**0.724±0.013**	0.754±0.011	**0**
	Standard - JSON	1	0.917	0.91	0.913	0.916	0.82	0.7	0.755	0
		2	0.85	0.835	0.841	0.848	0.777	0.722	0.749	0
		3	0.906	0.896	0.9	0.904	0.863	0.696	0.771	0
		Mean ± 95% CI	0.891±0.041	0.88±0.045	0.885±0.043	0.889±0.041	**0.82±0.049**	0.706±0.016	**0.758±0.013**	**0**
	CoT	1	0.896	0.881	0.887	0.892	0.823	0.656	0.73	0*
		2	0.898	0.887	0.892	0.896	0.746	0.7	0.723	0*
		3	0.889	0.879	0.883	0.888	0.562	0.683	0.616	0*
		Mean ± 95% CI	0.894±0.005	0.882±0.005	0.887±0.005	0.892±0.005	0.71±0.152	0.68±0.025	0.69±0.072	**0***
	Standard	1	0.906	0.905	0.905	0.908	0.575	0.727	0.642	0*
		2	0.901	0.892	0.896	0.9	0.773	0.674	0.72	0*
		3	0.9	0.894	0.896	0.9	0.593	0.714	0.648	0*
		Mean ± 95% CI	0.902±0.004	0.897±0.008	0.899±0.006	0.903±0.005	0.647±0.124	0.705±0.031	0.67±0.049	**0***
External Test	CoT - JSON	1	0.813	0.832	0.812	0.815	0.567	0.488	0.524	1
		2	0.779	0.796	0.768	0.77	0.596	0.446	0.51	1
		3	0.799	0.817	0.791	0.793	0.601	0.475	0.531	0
		Mean ± 95% CI	**0.797±0.019**	**0.815±0.02**	**0.79±0.025**	**0.793±0.025**	**0.588±0.021**	**0.47±0.024**	**0.522±0.012**	0-1
	Standard - JSON	1	0.788	0.803	0.771	0.772	0.602	0.439	0.508	3
		2	0.771	0.787	0.771	0.776	0.569	0.483	0.522	4
		3	0.797	0.813	0.783	0.785	0.567	0.455	0.505	3
		Mean ± 95% CI	0.785±0.015	0.801±0.015	0.775±0.008	0.778±0.008	0.579±0.022	0.459±0.025	0.512±0.01	3-4
	CoT	1	0.797	0.814	0.785	0.787	0.499	0.456	0.476	0*
		2	0.794	0.812	0.787	0.789	0.501	0.469	0.484	0*
		3	0.788	0.804	0.775	0.776	0.632	0.474	0.542	0*
		Mean ± 95% CI	0.793±0.005	0.81±0.006	0.782±0.007	0.784±0.008	0.544±0.086	0.466±0.011	0.501±0.041	**0***
	Standard	1	0.778	0.796	0.771	0.774	0.268	0.456	0.338	0*
		2	0.8	0.818	0.791	0.793	0.394	0.444	0.418	0*
		3	0.8	0.818	0.791	0.793	0.385	0.466	0.422	0*
		Mean ± 95% CI	0.793±0.014	0.811±0.014	0.784±0.013	0.787±0.012	0.349±0.08	0.455±0.012	0.393±0.054	**0***

Supplementary Table 3. Comparison of four prompting strategies on all LLMs trialed on the validation set. Best results for each model are underlined; best results overall are in bold. Results for a baseline GatorTron classification model are included at the top for reference.

Supplementary Table 4. Table showing all three seeds trained of chosen model for internal and external evaluation - Llama 3.1 8B with comparison of different prompt strategies. Values in bold represent best performance on that dataset.

			Document Level				Sentence Level			Parsing
	Prompt Strategy	Seed	Precision	Recall	F1	Accuracy	Precision	Recall	F1	Errors
Internal Test	Hybrid Decoding	1	0.928	0.923	0.925	0.928	0.779	0.714	0.745	0
		2	0.927	0.925	0.926	0.928	0.788	0.722	0.754	0
		3	0.902	0.9	0.901	0.904	0.795	0.736	0.764	0
		Mean ± 95% CI	0.919±0.017	**0.916±0.016**	**0.917±0.016**	**0.92±0.016**	0.787±0.009	**0.724±0.013**	0.754±0.011	**0**
	Greedy Decoding	1	0.928	0.923	0.925	0.928	0.779	0.714	0.745	0
		2	0.927	0.925	0.926	0.928	0.788	0.722	0.754	0
		3	0.902	0.9	0.901	0.904	0.795	0.736	0.764	0
		Mean ± 95% CI	0.919±0.017	**0.916±0.016**	**0.917±0.016**	**0.92±0.016**	0.787±0.009	**0.724±0.013**	0.754±0.011	**0**
	Nucleus Sampling	1	0.925	0.918	0.921	0.924	0.779	0.714	0.745	0
		2	0.923	0.92	0.921	0.924	0.776	0.718	0.746	0
		3	0.912	0.907	0.909	0.912	0.816	0.74	0.776	0
		Mean ± 95% CI	**0.92±0.008**	0.915±0.008	**0.917±0.008**	**0.92±0.008**	0.79±0.025	**0.724±0.016**	**0.756±0.02**	**0**
	Beam Search (4 Beams)	1	0.891	0.878	0.883	0.888	0.82	0.604	0.695	0
		2	0.894	0.892	0.893	0.896	0.772	0.643	0.702	0
		3	0.887	0.867	0.874	0.88	0.823	0.573	0.675	0
		Mean ± 95% CI	0.891±0.004	0.879±0.014	0.883±0.011	0.888±0.009	0.805±0.032	0.607±0.04	0.691±0.016	**0**
	Beam Search (8 Beams)	1	0.898	0.887	0.892	0.896	0.798	0.608	0.69	0
		2	0.893	0.893	0.893	0.896	0.8	0.687	0.739	0
		3	0.9	0.886	0.891	0.896	0.851	0.652	0.738	0
		Mean ± 95% CI	0.897±0.004	0.889±0.004	0.892±0.001	0.896±0	**0.816±0.034**	0.649±0.045	0.722±0.032	**0**
External Test	Hybrid Decoding	1	0.813	0.832	0.812	0.815	0.567	0.488	0.524	1
		2	0.779	0.796	0.768	0.77	0.596	0.446	0.51	1
		3	0.799	0.817	0.791	0.793	0.601	0.475	0.531	0
		Mean ± 95% CI	0.797±0.019	0.815±0.02	**0.79±0.025**	**0.793±0.025**	0.588±0.021	**0.47±0.024**	**0.522±0.012**	**0-1**
	Greedy Decoding	1	0.811	0.831	0.81	0.813	0.571	0.483	0.523	1
		2	0.779	0.796	0.768	0.77	0.596	0.446	0.51	1
		3	0.799	0.817	0.791	0.793	0.601	0.475	0.531	0
		Mean ± 95% CI	0.796±0.018	**0.815±0.02**	**0.79±0.024**	0.792±0.024	0.589±0.018	0.468±0.022	0.521±0.012	**0-1**
	Nucleus Sampling	1	0.809	0.828	0.806	0.809	0.562	0.476	0.516	0
		2	0.779	0.795	0.766	0.767	0.596	0.444	0.509	1
		3	0.806	0.824	0.796	0.798	0.598	0.47	0.526	0
		Mean ± 95% CI	**0.798±0.019**	**0.816±0.02**	0.789±0.024	0.791±0.025	0.585±0.023	0.463±0.019	0.517±0.01	**0-1**
	Beam Search (4 Beams)	1	0.763	0.778	0.749	0.75	0.673	0.383	0.488	0
		2	0.776	0.792	0.764	0.765	0.707	0.411	0.52	0
		3	0.781	0.789	0.75	0.75	0.72	0.383	0.5	0
		Mean ± 95% CI	0.773±0.011	0.786±0.008	0.754±0.009	0.755±0.01	**0.7±0.027**	0.392±0.018	0.503±0.018	**0**
	Beam Search (8 Beams)	1	0.785	0.803	0.776	0.778	0.67	0.399	0.5	0
		2	0.781	0.797	0.768	0.77	0.707	0.411	0.52	0
		3	0.796	0.81	0.775	0.776	0.711	0.406	0.517	0
		Mean ± 95% CI	0.787±0.009	0.803±0.007	0.773±0.005	0.775±0.005	0.696±0.026	0.405±0.007	0.512±0.012	**0**

Supplementary Table 5. Table showing all three seeds trained of chosen model for internal and external evaluation - Llama 3.1 8B with comparison of different prompt strategies. Values in bold represent best performance on that dataset.

10 Gatortron Baseline Methodology Details

For this baseline encoder-only non-generative language model we follow the standard procedure to fine-tune a sequence classification BERT style model using Huggingface/Transformers. The base language model can be found here: https://huggingface.co/UFNLP/gatortron-base. The model is trained to take a sentence and classify whether it is an AIF or not. PET-CT reports are usually longer than the 512 token limit for BERT style models (GatorTron included) so sentence level classification is required. The report is tokenized into sentences using the same sentence tokenizer used for the generative models. At inference time the report is split into sentences, the fine-tuned GatorTron model classifies each sentence, and positive classifications are added to the AIF list. One or more AIFs found in a report is considered a positive report at document level for evaluation purposes.

In terms of hyperparameters we used a linearly decaying learning rate of 1e-5. A batch size of eight and trained for five epochs. This was found to offer the best performance on the validation set sentences.

11 Model Quantization Comparison

This experiment compared LoRA fine-tuning of Llama 3.1/3.2 1B, 3B and 8B models when using the full precision 16-bit models against QLoRA 4-bit models on the internal test dataset. We found no significant difference in performance that would justify using 16-bit models over 4-bit models for the main project, if anything the quantised models were slightly better. Please note slightly different hyperparameter settings were used on this experiment as they were still being tuned for the final experiments.

Model	Model Encoding	Precision	Recall	F1	Accuracy	Precision	Recall	F1
Llama-3.1-8b-Instruct	4-bit	**0.917**	**0.919**	**0.918**	**0.92**	**0.801**	**0.727**	**0.762**
Llama-3.1-8b-Instruct	16-bit	0.897	0.898	0.897	0.9	0.757	0.7	0.728
Llama-3.2-3b-Instruct	4-bit	0.883	0.877	0.88	**0.884**	**0.727**	**0.634**	**0.678**
Llama-3.2-3b-Instruct	16-bit	**0.897**	**0.887**	**0.882**	**0.884**	0.65	0.63	0.64
Llama-3.2-1b-Instruct	4-bit	**0.846**	**0.839**	**0.842**	**0.848**	**0.53**	**0.422**	**0.471**
Llama-3.2-1b-Instruct	16-bit	0.838	0.831	0.834	0.84	0.458	0.388	0.42

Supplementary Table 6. Table showing the effect of model quantization on three Llama models. Bold values represent if the 4-bit or 16-bit version achieved a higher score.

12 Supplementary Material References

1. Giarelis N, Mastrokostas C, Karacapilidis N. Abstractive Vs. Extractive Summarization: An Experimental Review. *Applied Sciences* (2023) 13(13):7620.

2. Papineni K, Roukos S, Ward T, Zhu W-J, editors. Bleu: A Method for Automatic Evaluation of Machine Translation. *Proceedings of the 40th annual meeting of the Association for Computational Linguistics*; 2002.

3. Lin C-Y, editor. Rouge: A Package for Automatic Evaluation of Summaries. *Text summarization branches out*; 2004.

4. Tay W, Joshi A, Zhang XJ, Karimi S, Wan S, editors. Red-Faced Rouge: Examining the Suitability of Rouge for Opinion Summary Evaluation. *Proceedings of the 17th annual workshop of the Australasian language technology association*; 2019.

5. Sulem E, Abend O, Rappoport A. Bleu Is Not Suitable for the Evaluation of Text Simplification. *arXiv preprint arXiv:181005995* (2018).

6. Bird S, Loper E, editors. Nltk: The Natural Language Toolkit. 2004 July; Barcelona, Spain: Association for Computational Linguistics.

7. Vaswani A, Shazeer N, Parmar N, Uszkoreit J, Jones L, Gomez AN, et al. Attention Is All You Need. *Advances in neural information processing systems* (2017) 30.

8. Li Y, Bubeck S, Eldan R, Del Giorno A, Gunasekar S, Lee YT. Textbooks Are All You Need Ii: Phi-1.5 Technical Report. *arXiv preprint arXiv:230905463* (2023).

9. Grattafiori A, Dubey A, Jauhri A, Pandey A, Kadian A, Al-Dahle A, et al. The Llama 3 Herd of Models. *arXiv preprint arXiv:240721783* (2024).

10. Dettmers T, Pagnoni A, Holtzman A, Zettlemoyer L. Qlora: Efficient Finetuning of Quantized Llms. *Advances in Neural Information Processing Systems* (2024) 36.

11. Han DH, Michael; Unsloth team. *Unsloth*. (2023).
